# Treatment with the EkoSonic™ Endovascular System (EKOS^®^): initial experience in Brazil and technical tips and tricks - a case series report

**DOI:** 10.36416/1806-3756/e20250260

**Published:** 2026-03-05

**Authors:** Túlio Fabiano de Oliveira Leite, Daladie Rodrigues Parreira

**Affiliations:** 1. Radiology, Túlio Radiologia Intervencionista Ltda., Uberlândia (MG), Brasil.; 2. Cardiac Surgery, Universidade Federal de Uberlândia, Uberlândia (MG), Brasil.

## TO THE EDITOR,

Pulmonary embolism (PE) is a serious obstructive cardiovascular condition. Among cardiovascular disorders, it is the third most common acute syndrome.^1^ In Brazil, data on PE remain scarce: from 2008 to 2019, 81,152 cases were reported, with mortality rates of up to 21.21%.^2^ PE has numerous known causes, and current treatment guidelines recommend hospitalization and systemic anticoagulation; however, mild cases may be managed in an outpatient setting. The objective of this study was to describe the safety and efficacy of thrombolysis using the EkoSonic™ endovascular system (EKOS) in patients with PE in Brazil.

Several modalities of catheter-directed treatment (CDT) are available, including large-bore mechanical thrombectomy (MT) and ultrasound-assisted catheter-directed thrombolysis (USAT). The EKOS system consists of a 5.4-Fr multilumen catheter for the infusion of thrombolytic agents directly into the pulmonary arteries and an ultrasonic core containing multiple high-frequency (1.5-1.9 MHz), low-power (0.5 W per element) transducers.

A total of 10 patients with acute PE treated between June 2023 and January 2025 in a private hospital in Brazil were retrospectively reviewed. Written informed consent was obtained from all patients, or from an authorized family member, before port implantation. Patients were selected based on clinical indication, with diagnosis confirmed by pulmonary CT angiography and echocardiography. The simplified Pulmonary Embolism Severity Index (PESI) was calculated to estimate the 30-day mortality risk and to identify low-risk PE patients who could be managed without hospital admission. 

Treatment decisions were based on consensus from a multidisciplinary Pulmonary Embolism Response Team (PERT) for patients with massive and submassive PE. There were no exclusion criteria for the use of EKOS or rtPA. All patients received EKOS therapy, and system implantation was performed in the interventional radiology suite. All patients were anticoagulated with enoxaparin 1.0 mg/kg subcutaneously every 12 hours. 

**Figure 1:**
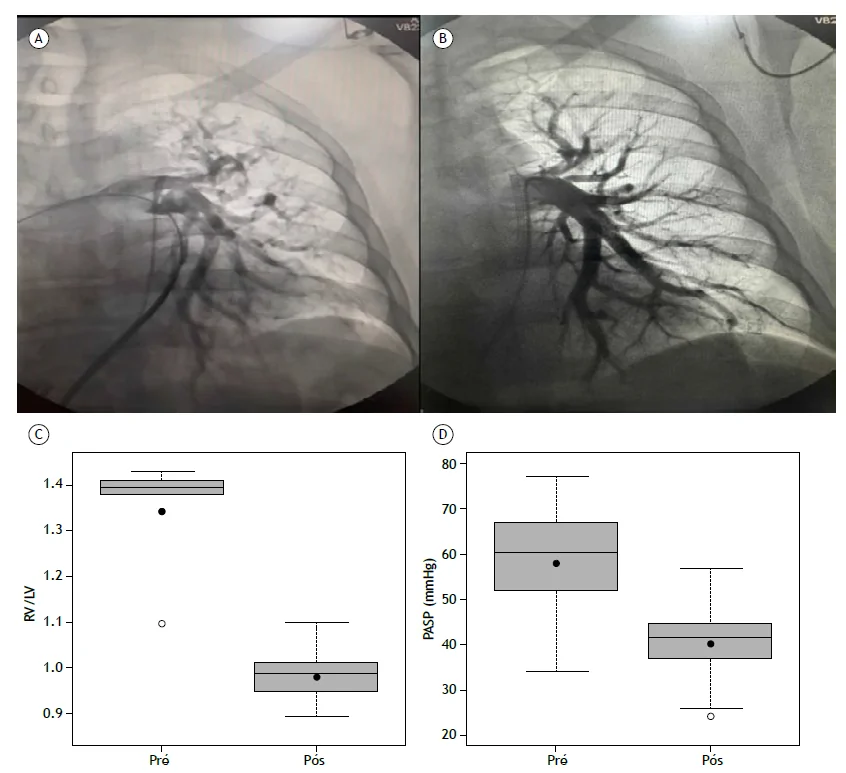
(A) Left pulmonary artery angiography confirming near-complete obstruction at the origin. (B) Left pulmonary artery angiography after treatment with EKOS, showing improved arterial perfusion. (C) Boxplot of the RV/LV ratio before and after the procedure (RV/LV: right-to-left ventricular diameter ratio). (D) Boxplot of pulmonary artery systolic pressure (PASP) before and after the procedure.

Femoral access was obtained under strict sterile-barrier precautions, with sedation and local anesthesia; 2% lidocaine was administered under ultrasound guidance. Two 6-Fr, 65-cm introducers were inserted under direct ultrasound guidance into the bilateral femoral veins. Pulmonary arteriography and pulmonary artery pressure (PAP) measurements were performed before positioning the EKOS system. Recombinant tissue plasminogen activator (rtPA) was infused at a rate of 1.0 mg/catheter/h, along with saline at 35 mL/h. After EKOS system placement, all patients were transferred to the intensive care unit (Figure 1).

The change in the ratio between the right and left ventricular diameters (RV/LV) on transthoracic echocardiography, as well as the change in PAP relative to baseline, was evaluated before the procedure and 48 hours afterward.

After a detailed description of the variables of interest, a linear mixed-effects model was fitted using a Bayesian approach to compare the mean values of PAP and the RV/LV ratio between the pre- and post-procedure time points. These models included a random effect to account for intra-individual variability, i.e., the dependence between repeated measurements from the same patient at both time points. Mean differences and corresponding 95% credibility intervals (95% CIs) were estimated. The analyses were performed using the R2jags package in R version 4.3.2.

Patient clinical and demographic characteristics are presented in Table 1. During the study period, 10 patients were diagnosed with submassive or massive PE and underwent thrombolysis with the EKOS system. The mean rtPA dose was 24.60 mg (range, 14-30 mg), and the mean infusion time was 12.5 h (range, 10-14 h). 

**Table 1:** Demographic and clinical characteristics and treatment details of the study population.

Characteristics	Value*
Age (years [mean and range])	60.7 (34-90)
Sex	
Male	8 (80%)
Female	2 (20%)
Ethnicity	
Black	3 (30%)
White	7 (70%)
BMI	27.88 (22.91-35.43)
Laterality	
Bilateral	10 (100%)
No. of treatments	10 (100%)
PE Subtype	
Massive	5 (50%)
Submassive	5 (50%)
sPESI	3.10 (2-4)
Time of diagnosis to procedure (h)	18.40 (10-28)
Post-treatment imaging	
Angiography (No. of patients)	10 (100%)
Echocardiography (No. of patients)	10 (100%)
RV pressures (mmHg [mean and range])	
Before treatment	58 (34-77)
After treatment	40.5 (24-57)
Thrombolytic dose (mg [mean and range])	24.60 (14-30)
Saline solution COOLANT (mL [mean and range])	420 (350-490)
Infusion time (h [mean and range])	12.5 (10-14)
ICU stay (days [mean and range])	3.4 (1-10)
Hospital stay (days [media])	7.5 (3-30)

sPESI, simplified Pulmonary Embolism Severity Index; h, hour(s); ICU, Intensive Care Unit; BMI, Body Mass Index; PE, Pulmonary Embolism; RV, right ventricular. * = Averages and 95% credibility intervals (95% CIs), calculated using a Bayesian approach.

Pre-treatment RV pressures were compared with post-treatment values. The mean RV pressure before intervention was 58.10 mmHg (range, 34-77 mmHg), which decreased to 40.5 mmHg (range, 24-57 mmHg) after thrombolysis with the EKOS system. The mean RV/LV ratio before intervention was 1.34 (range, 1.10-1.43), decreasing to 0.98 (range, 0.89-1.10) after treatment (Figure 1). 

No bleeding complications occurred. One death was recorded, attributed to underlying gastric neoplasia and unrelated to the EKOS system.

Interventions in PE cases aim to achieve more efficient and early recanalization to restore distal flow. PE treatment is anchored in five pillars: restoring perfusion, ensuring hemodynamic stability, enabling adequate tissue oxygenation, preventing disease recurrence, and facilitating multidisciplinary management. Hemodynamic instability and impaired tissue oxygenation can be managed with volume optimization, vasopressors, inotropes, extracorporeal membrane oxygenation, and ventilatory support when necessary.^1^

Several studies have demonstrated that systemic thrombolytic therapy leads to faster improvements in PAP, reductions in pulmonary vascular resistance (PVR), and decreases in RV dilation; however, it is associated with an increased risk of extracranial bleeding (9.9%) and major intracranial bleeding (1.7%) compared to anticoagulation alone.^4^

The objective of the EKOS system is to fragment fibrin using high-frequency ultrasonic energy combined with the low-dose infusion of a fibronolytic agent, thereby maximizing the thrombus surface area and optimizing the dose-effect relationship of thrombolytic therapy. The SEATTLE II, ULTIMA, OPTALYSE, and PERFECT trials demonstrated that the use of EKOS in patients with massive and submassive PE resulted in symptomatic and clinical improvement, reductions in the RV/LV ratio, decreased PAP, and minimal complications.^5^ ^,^ ^6^ ^,^ ^7^ ^,^ ^8^

MT and USAT have demonstrated similar technical success rates (99.6% vs. 99.4%) in the treatment of PE. MT was associated with longer procedure times, lower blood loss, and shorter ICU and overall hospital stays. EKOS showed a greater mean change in the Miller Index, as well as a greater reduction in PAP. Therefore, devices with different technologies can produce comparable outcomes in patients with pulmonary embolism.^9^

One patient died five months after discharge due to complications related to gastric neoplasia. Moreover, our findings-using a mean rtPA dose of 24.6 mg per patient and a mean infusion time of 18.4 h, with improvements in the RV/LV ratio and reductions in PAP-are consistent with those reported in the literature.^5^ ^,^ ^6^ ^,^ ^7^ ^,^ ^8^

Throughout our experience with the EKOS system, we observed that an infusion rate of 1.0 to 1.5 mg/h/catheter, depending on the patient’s clinical characteristics and the extent of their pulmonary thrombotic burden, can achieve satisfactory results. Furthermore, we found that using the smallest possible volume of saline solution in the COOLANT (35 mL/h) is sufficient to maintain proper device functioning. This is important because excessive saline infusion can overload the cardiopulmonary system, leading to pulmonary edema or ventricular strain.

The PEERLESS study is an ongoing randomized controlled trial comparing large-bore mechanical thrombectomy using the FlowTriever system (Inari Medical, Irvine, CA, USA) with conventional catheter-directed thrombolysis and EKOS-guided thrombolysis. The objective of that study is to provide data to support the selection of treatment strategies after the decision to intervene has been made.^10^ Therefore, the PEERLESS trial will be important in determining which device is most suitable for each type of patient with PE.

The main limitations of our study were the small patient population, its single-center retrospective design, and the lack of comparison with other endovascular devices. Although the sample size was small, we were able to evaluate and demonstrate a significant improvement in cardiac function, reflected by reductions in the RV/LV ratio and PAP, without complications. Thus, randomized studies comparing mechanical thrombectomy (MT) and ultrasound-assisted catheter-directed thrombolysis (USAT) are needed to determine which patients would benefit most from these techniques.

The EKOS system improves the RV/LV ratio and reduces PAP, with low complication rates. This study expands the experience with low-dose fibrinolysis in patients with PE in Brazil and demonstrates the potential applicability of this device.

## References

[1] Konstantinides SV, Meyer G, Becattini C, Bueno H, Geersing GJ, Harjola VP (2020). 2019 ESC Guidelines for the diagnosis and management of acute pulmonary embolism developed in collaboration with the European Respiratory Society (ERS). Eur Heart J.

[2] Gomes JA, Barros JEB, Nascimento ALOD, Rocha CAO, Almeida JPO, Santana GBA (2022). Hospitalizations for pulmonary embolism in Brazil (2008-2019) an ecological and time series study. J Bras Pneumol.

[3] Rivera-Lebron BN, Rali PM, Tapson VF (2021). The PERT Concept A Step-by-Step Approach to Managing Pulmonary Embolism. Chest.

[4] Mathew D, Seelam S, Bumrah K, Sherif A, Shrestha U (2023). Systemic thrombolysis with newer thrombolytics vs anticoagulation in acute intermediate risk pulmonary embolism a systematic review and meta-analysis. BMC Cardiovasc Disord.

[5] Piazza G, Hohlfelder B, Jaff MR, Ouriel K, Engelhardt TC, Sterling KM (2015). A Prospective, Single-Arm, Multicenter Trial of Ultrasound-Facilitated, Catheter-Directed, Low-Dose Fibrinolysis for Acute Massive and Submassive Pulmonary Embolism The SEATTLE II Study. JACC Cardiovasc Interv.

[6] Kucher N, Boekstegers P, Müller OJ, Kupatt C, Beyer-Westendorf J, Heitzer T (2014). Randomized, controlled trial of ultrasound-assisted catheter-directed thrombolysis for acute intermediate-risk pulmonary embolism. Circulation.

[7] Tapson VF, Sterling K, Jones N, Elder M, Tripathy U, Brower J (2018). A Randomized Trial of the Optimum Duration of Acoustic Pulse Thrombolysis Procedure in Acute Intermediate-Risk Pulmonary Embolism The OPTALYSE PE Trial. JACC Cardiovasc Interv.

[8] Kuo WT, Banerjee A, Kim PS, DeMarco FJ, Levy JR, Facchini FR (2015). Pulmonary Embolism Response to Fragmentation, Embolectomy, and Catheter Thrombolysis (PERFECT) Initial Results From a Prospective Multicenter Registry. Chest.

[9] Choksi EJ, Sare A, Shukla PA, Kumar A (2025). Comparison of Safety and Efficacy of Aspiration Thrombectomy and Ultrasound Accelerated Thrombolysis for Management of Pulmonary Embolism A Systematic Review and Meta-Analysis. Vasc Endovascular Surg.

[10] Gonsalves CF, Gibson CM, Stortecky S, Alvarez RA, Beam DM, Horowitz JM (2023). Randomized controlled trial of mechanical thrombectomy vs catheter-directed thrombolysis for acute hemodynamically stable pulmonary embolism Rationale and design of the PEERLESS study. Am Heart J.

